# Crystal structure of 2-(1*H*-imidazol-4-yl)ethanaminium chloride

**DOI:** 10.1107/S2056989015006866

**Published:** 2015-04-09

**Authors:** Imene Belfilali, Siham Yebdri, Samira Louhibi, Leila Boukli-hacene, Thierry Roisnel

**Affiliations:** a Laboratoire de Chimie Inorganique et Environnement, University of Tlemcen, BP 119, 13000, Tlemcen, Algeria; b Centre de Diffractometrie X, UMR 6226 CNRS, Unite Sciences Chimiques de Rennes, Universite de Rennes I, 263 Avenue du General Leclerc, 35042 Rennes, France

**Keywords:** crystal structure, histamine, imidazole, chloride Ion, protonation, hydrogen bonding

## Abstract

The title mol­ecular salt, C_5_H_10_N_3_^+^·Cl^−^, was obtained as by-product in the attempted synthesis of a histamine derivative. The terminal amino group of the starting material is protonated. The C_imidazole_—C—C—N(H_3_)^+^ group in the cation is in an *anti* conformation with a torsion angle of 176.22 (10)°. In the crystal, cations and anions are linked *via* N—H⋯N and N—H—Cl hydrogen bonds, forming a two-dimensional network parallel to (10-1). A single weak C—H⋯Cl hydrogen bond completes a three-dimensional network.

## Related literature   

For the biological and pharmacological applications of histamine derivatives, see: Barnes *et al.* (2001 ▸); Schwartz *et al.* (1991 ▸); Bachert *et al.* (1998 ▸); Emanuel *et al.* (1999 ▸); Apáti *et al.* (2012 ▸). For a study of a histamine copper(II) chloride complex, see: Belfilali *et al.* (2015 ▸). For the general chemistry of transition metal ions with histamine, see: Mikulski *et al.* (2012 ▸); Kowalik-Jankowska *et al.* (2010 ▸); Selmeczi *et al.* (2012 ▸). For a related structure, see: Prout *et al.* (1974 ▸).
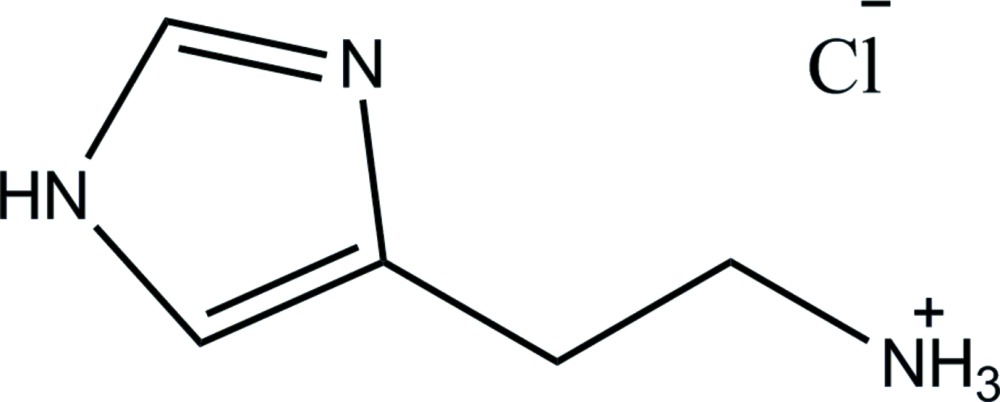


## Experimental   

### Crystal data  

C_5_H_10_N_3_^+^·Cl^−^
*M* *_r_* = 147.61
Monoclinic, 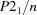

*a* = 4.5840 (2) Å
*b* = 9.1614 (3) Å
*c* = 17.3114 (5) Å
β = 91.682 (1)°
*V* = 726.69 (4) Å^3^
*Z* = 4
Mo *K*α radiation
μ = 0.44 mm^−1^
*T* = 150 K
0.41 × 0.13 × 0.08 mm

### Data collection  

Bruker APEXII diffractometer
Absorption correction: multi-scan (*SADABS*; Bruker, 2006 ▸)’ *T*_min_ = 0.868, *T*_max_ = 0.965
5568 measured reflections
1645 independent reflections
1494 reflections with *I* > 2σ(*I*)
*R*_int_ = 0.033

### Refinement  

*R*[*F* ^2^ > 2σ(*F* ^2^)] = 0.028
*wR*(*F* ^2^) = 0.076
*S* = 1.08
1645 reflections
86 parameters
H atoms treated by a mixture of independent and constrained refinement
Δρ_max_ = 0.34 e Å^−3^
Δρ_min_ = −0.21 e Å^−3^

### 

Data collection: *APEX2* (Bruker, 2006 ▸); cell refinement: *SAINT* (Bruker, 2006 ▸); data reduction: *SAINT*; program(s) used to solve structure: *SIR97* (Altomare *et al.*, 1999 ▸); program(s) used to refine structure: *SHELXL97* (Sheldrick, 2008 ▸); molecular graphics: *ORTEP-3 for Windows* (Farrugia, 2012 ▸) and *PLATON* (Spek, 2009 ▸); software used to prepare material for publication: *WinGX* publication routines (Farrugia, 2012 ▸) and *CRYSCAL* (T. Roisnel, local program).

## Supplementary Material

Crystal structure: contains datablock(s) I. DOI: 10.1107/S2056989015006866/lh5756sup1.cif

Structure factors: contains datablock(s) I. DOI: 10.1107/S2056989015006866/lh5756Isup2.hkl

Supporting information file. DOI: 10.1107/S2056989015006866/lh5756Isup3.docx

**Fig. 1.**
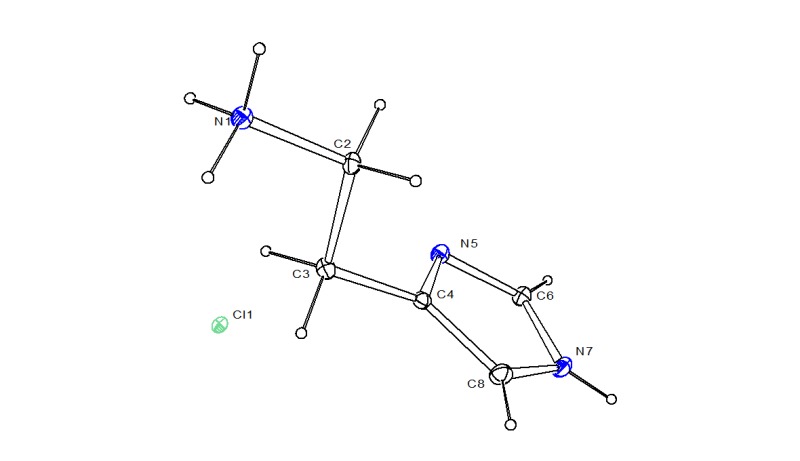
The molecular structure of the title compound. Displacement ellipsoids are drawn at the 50% probability level.

. DOI: 10.1107/S2056989015006866/lh5756fig1.tif
The mol­ecular structure of the title compound. Displacement ellipsoids are drawn at the 50% probability level.

**Fig. 2.**
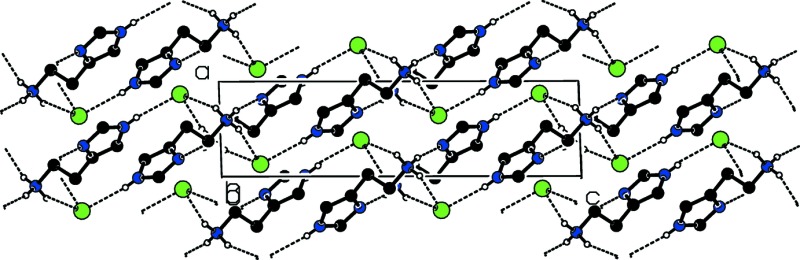
Part of the crystal structure with hydrogen bonds shown as dashed lines.

. DOI: 10.1107/S2056989015006866/lh5756fig2.tif
Part of the crystal structure with hydrogen bonds shown as dashed lines.

CCDC reference: 1051527

Additional supporting information:  crystallographic information; 3D view; checkCIF report

## Figures and Tables

**Table 1:** Hydrogen-bond geometry (, )

*D*H*A*	*D*H	H*A*	*D* *A*	*D*H*A*
N1H1*A*N5^i^	0.91	1.96	2.8508(15)	168
N1H1*B*Cl1^i^	0.91	2.28	3.1557(11)	160
N1H1*C*Cl1^ii^	0.91	2.39	3.2443(11)	157
N7H7Cl1^iii^	0.78(2)	2.40(2)	3.1645(12)	168(2)
C2H2*A*Cl1^iv^	0.99	2.72	3.6974(14)	168

Symmetry codes: (i) 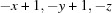; (ii) 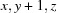; (iii) 
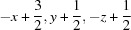
; (iv) 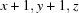.

## supplementary crystallographic information

### S1. Structural commentary

Histamine (2-(1H-imidazol-4-yl)ethanamine) is a biogenic amine present in essentially all mammalian tissues and involved in several defense mechanisms of the body. It plays a role in various physiological processes, such as control of gastric acid secretion, neurotransmission, regulation of the microcirculation, and modulation of inflammatory (Barnes *et al.*, 2001) and immunological reactions (Schwartz *et al.*, 1991; Bachert *et al.*, 1998; Emanuel *et al.*, 1999) as well as its uses in pharmacology (Apáti *et al.*, 2012). Moreover, the inter­action of transition metal ions with histamine (Mikulski *et al.*, 2012), play a key role in catalysis processes (Kowalik-Jankowska *et al.*, 2010; Selmeczi *et al.*, 2012). We have previously reported the preparation and the crystal structure of the histamine copper(II) chloride complex and its catalytic activity study (Belfilali *et al.*, 2015). In this study, we report the synthesis and crystal structure determination of the title compound.

The molecular structure of the title compound is shown in Fig. 1. The organic cation displays a trans conformation with respect to the amine group and the imidazole ring about the –CH_2_—CH_2_– bond of the side chain with a torsion angle of 176.22 (10)° for N1–C2–C3–C4. The bond lengths and angles are within normal ranges and are comparable to a related structure (Prout *et al.*, 1974). In the crystal, cations and anions are linked via N—H···N and N—H—Cl hydrogen bonds two form a two-dimensional network (Fig. 2) parallel to (101). A single weak C—H···Cl hydrogen bond completes a three-dimensinal network.

### S2. Synthesis and crystallization

A mixture of histamine di­hydro­chloride (1.0 mmol) and methyl-1hy­droxy-2-naphtho­ate (1 mmol) were taken in a beaker placed in a microwave oven and irradiated at 200 watt for 5 minutes. After completion the reaction, the reaction mixture was allowed to reach room temperature and the resulting crystals were separated by filtration.

### S3. Refinement

H atoms bonded to C atoms were included in calculated positions with C—H = 0.95 – 0.99 Å and U_iso_(H) = 1.2U_eq_(C). H atoms bonded to N1 were included in calculated positions with N—H = 0.91Å and U_iso_(H) = 1.5U_eq_(N). The H atom bonded to N7 was refined independently with an isotropic displacement parameter.

### Crystal data

**Table:**

C_5_H_10_N_3_^+^·Cl^−^	*F*(000) = 312
*M**_r_* = 147.61	*D*_x_ = 1.349 Mg m^−^^3^
Monoclinic, *P*2_1_/*n*	Mo *K*α radiation, λ = 0.71073 Å
Hall symbol: -P 2yn	Cell parameters from 2978 reflections
*a* = 4.5840 (2) Å	θ = 4.6–27.5°
*b* = 9.1614 (3) Å	µ = 0.44 mm^−^^1^
*c* = 17.3114 (5) Å	*T* = 150 K
β = 91.682 (1)°	Prism, colourless
*V* = 726.69 (4) Å^3^	0.41 × 0.13 × 0.08 mm
*Z* = 4	

### Data collection

**Table:**

Bruker APEXII diffractometer	1494 reflections with *I* > 2σ(*I*)
Graphite monochromator	*R*_int_ = 0.033
CCD rotation images, thin slices scans	θ_max_ = 27.5°, θ_min_ = 3.2°
Absorption correction: multi-scan (*SADABS*; Bruker, 2006)'	*h* = −5→5
*T*_min_ = 0.868, *T*_max_ = 0.965	*k* = −11→11
5568 measured reflections	*l* = −19→22
1645 independent reflections	

### Refinement

**Table:**

Refinement on *F*^2^	Primary atom site location: structure-invariant direct methods
Least-squares matrix: full	Secondary atom site location: difference Fourier map
*R*[*F*^2^ > 2σ(*F*^2^)] = 0.028	Hydrogen site location: inferred from neighbouring sites
*wR*(*F*^2^) = 0.076	H atoms treated by a mixture of independent and constrained refinement
*S* = 1.08	*w* = 1/[σ^2^(*F*_o_^2^) + (0.0331*P*)^2^ + 0.246*P*] where *P* = (*F*_o_^2^ + 2*F*_c_^2^)/3
1645 reflections	(Δ/σ)_max_ = 0.001
86 parameters	Δρ_max_ = 0.34 e Å^−^^3^
0 restraints	Δρ_min_ = −0.21 e Å^−^^3^

### Special details

**Table:**

Geometry. All e.s.d.'s (except the e.s.d. in the dihedral angle between two l.s. planes) are estimated using the full covariance matrix. The cell e.s.d.'s are taken into account individually in the estimation of e.s.d.'s in distances, angles and torsion angles; correlations between e.s.d.'s in cell parameters are only used when they are defined by crystal symmetry. An approximate (isotropic) treatment of cell e.s.d.'s is used for estimating e.s.d.'s involving l.s. planes.
Refinement. Refinement of *F*^2^ against ALL reflections. The weighted *R*-factor *wR* and goodness of fit *S* are based on *F*^2^, conventional *R*-factors *R* are based on *F*, with *F* set to zero for negative *F*^2^. The threshold expression of *F*^2^ > σ(*F*^2^) is used only for calculating *R*-factors(gt) *etc*. and is not relevant to the choice of reflections for refinement. *R*-factors based on *F*^2^ are statistically about twice as large as those based on *F*, and *R*- factors based on ALL data will be even larger.

### Fractional atomic coordinates and isotropic or equivalent isotropic displacement parameters (Å^2^)

**Table:**

	*x*	*y*	*z*	*U*_iso_*/*U*_eq_	
N1	0.4106 (2)	0.80020 (12)	−0.01832 (6)	0.0174 (2)	
H1A	0.3108	0.7313	−0.0462	0.026*	
H1B	0.5029	0.8614	−0.051	0.026*	
H1C	0.2838	0.8521	0.0104	0.026*	
C2	0.6308 (3)	0.72762 (15)	0.03395 (7)	0.0174 (3)	
H2A	0.7497	0.8028	0.0613	0.021*	
H2B	0.7631	0.6675	0.0029	0.021*	
C3	0.4835 (3)	0.63146 (15)	0.09277 (8)	0.0192 (3)	
H3A	0.3615	0.6926	0.1262	0.023*	
H3B	0.3543	0.5605	0.0655	0.023*	
C4	0.7051 (3)	0.55095 (15)	0.14180 (7)	0.0169 (3)	
N5	0.8253 (2)	0.41984 (12)	0.11844 (6)	0.0184 (2)	
C6	1.0162 (3)	0.38371 (15)	0.17436 (7)	0.0197 (3)	
H6	1.1334	0.2982	0.1741	0.024*	
N7	1.0229 (3)	0.48288 (14)	0.23111 (7)	0.0223 (3)	
H7	1.124 (5)	0.481 (2)	0.2682 (14)	0.05*	
C8	0.8270 (3)	0.58985 (16)	0.21139 (8)	0.0229 (3)	
H8	0.7842	0.6747	0.2405	0.027*	
Cl1	0.13058 (7)	0.01456 (3)	0.108958 (17)	0.01841 (12)	

### Atomic displacement parameters (Å^2^)

**Table:**

	*U*^11^	*U*^22^	*U*^33^	*U*^12^	*U*^13^	*U*^23^
N1	0.0185 (5)	0.0155 (6)	0.0180 (5)	−0.0019 (4)	−0.0008 (4)	−0.0004 (4)
C2	0.0152 (6)	0.0180 (6)	0.0190 (6)	−0.0021 (5)	−0.0022 (5)	−0.0002 (5)
C3	0.0156 (6)	0.0200 (7)	0.0220 (7)	−0.0008 (5)	0.0007 (5)	0.0009 (5)
C4	0.0160 (6)	0.0180 (6)	0.0169 (6)	−0.0017 (5)	0.0031 (5)	0.0006 (5)
N5	0.0206 (5)	0.0155 (5)	0.0189 (5)	−0.0016 (4)	−0.0018 (4)	−0.0006 (5)
C6	0.0218 (6)	0.0176 (6)	0.0197 (6)	−0.0007 (5)	−0.0012 (5)	0.0015 (5)
N7	0.0238 (6)	0.0268 (6)	0.0161 (6)	0.0001 (5)	−0.0041 (5)	−0.0009 (5)
C8	0.0252 (7)	0.0233 (7)	0.0202 (7)	0.0034 (6)	0.0008 (5)	−0.0046 (6)
Cl1	0.02061 (18)	0.01856 (18)	0.01589 (19)	−0.00105 (12)	−0.00236 (12)	−0.00087 (11)

### Geometric parameters (Å, º)

**Table:**

N1—C2	1.4920 (16)	C3—H3B	0.99
N1—H1A	0.91	C4—C8	1.3604 (18)
N1—H1B	0.91	C4—N5	1.3866 (17)
N1—H1C	0.91	N5—C6	1.3277 (16)
C2—C3	1.5196 (18)	C6—N7	1.3379 (18)
C2—H2A	0.99	C6—H6	0.95
C2—H2B	0.99	N7—C8	1.3658 (18)
C3—C4	1.4985 (18)	N7—H7	0.78 (2)
C3—H3A	0.99	C8—H8	0.95
			
C2—N1—H1A	109.5	C2—C3—H3B	109.4
C2—N1—H1B	109.5	H3A—C3—H3B	108
H1A—N1—H1B	109.5	C8—C4—N5	109.20 (11)
C2—N1—H1C	109.5	C8—C4—C3	128.79 (13)
H1A—N1—H1C	109.5	N5—C4—C3	121.99 (11)
H1B—N1—H1C	109.5	C6—N5—C4	105.21 (11)
N1—C2—C3	111.03 (10)	N5—C6—N7	111.50 (12)
N1—C2—H2A	109.4	N5—C6—H6	124.2
C3—C2—H2A	109.4	N7—C6—H6	124.2
N1—C2—H2B	109.4	C6—N7—C8	107.63 (11)
C3—C2—H2B	109.4	C6—N7—H7	126.3 (16)
H2A—C2—H2B	108	C8—N7—H7	126.1 (16)
C4—C3—C2	110.96 (10)	C4—C8—N7	106.46 (12)
C4—C3—H3A	109.4	C4—C8—H8	126.8
C2—C3—H3A	109.4	N7—C8—H8	126.8
C4—C3—H3B	109.4		
			
N1—C2—C3—C4	176.22 (10)	C4—N5—C6—N7	0.21 (15)
C2—C3—C4—C8	93.03 (17)	N5—C6—N7—C8	−0.18 (16)
C2—C3—C4—N5	−84.87 (15)	N5—C4—C8—N7	0.06 (15)
C8—C4—N5—C6	−0.16 (15)	C3—C4—C8—N7	−178.06 (12)
C3—C4—N5—C6	178.11 (12)	C6—N7—C8—C4	0.07 (16)

### Hydrogen-bond geometry (Å, º)

**Table:**

*D*—H···*A*	*D*—H	H···*A*	*D*···*A*	*D*—H···*A*
N1—H1*A*···N5^i^	0.91	1.96	2.8508 (15)	168
N1—H1*B*···Cl1^i^	0.91	2.28	3.1557 (11)	160
N1—H1*C*···Cl1^ii^	0.91	2.39	3.2443 (11)	157
N7—H7···Cl1^iii^	0.78 (2)	2.40 (2)	3.1645 (12)	168 (2)
C2—H2*A*···Cl1^iv^	0.99	2.72	3.6974 (14)	168

Symmetry codes: (i) −*x*+1, −*y*+1, −*z*; (ii) *x*, *y*+1, *z*; (iii) −*x*+3/2, *y*+1/2, −*z*+1/2; (iv) *x*+1, *y*+1, *z*.
