# Supplementary material for: Crystal structure of 2-(1H-imidazol-4-yl)ethanaminium chloride
Source: Acta Crystallogr E Crystallogr Commun. 2015 Apr 9;71(Pt 5):o301–2. doi: 10.1107/S2056989015006866 (PMC4420062; doi:10.1107/S2056989015006866)
Supplement: Supplementary file 3 [file e-71-0o301-Isup3.docx]

**supporting informations**

**S1- Comment**

Histamine (2-(1H-imidazol-4-yl)ethanamine) is a biogenic amine present in essentially all mammalian tissues and involved in several defense mechanisms of the body. It plays a role in various physiological processes, such as control of gastric acid secretion, neurotransmission, regulation of the microcirculation, and modulation of inflammatory (Barnes et al., 2001) and immunological reactions ( Schwartz et al., 1991 ; Bachert et al., 1998 ; Emanuel et al., 1999 ).

We have previously reported the preparation, the crystal structure of the histamine copper(II) chloride complex and its catalytic activity study (Belfilali et al., 2015). ). In this study, we report the synthesis of histamine monoprotonated form, 2-(1H-imidazol-4-yl)ethanaminium chloride and its crystal structure.

The molecular structure of the title compound (Fig. 1), has a chair form. The bond lengths and angles are within normal ranges and are comparable to related structure (Houari et al., 2013). The chloride ion is the center of many hydrogen bonds forming a 3D network, as shown in Fig. 2 and table 1.

**S2. Experimental**

A mixture of histamine dihydrochloride (1.0 mmol) and methyl-1hydroxy-2-naphthoate (1 mmol) were taken in a beaker placed in a microwave oven and irradiated at 200 watt for 5 minutes. After completion the reaction, the reaction mixture was allowed to attain room temperature and crystals separated by filtration.

**S3. Refinement**

All non-hydrogen atoms were refined with anisotropic atomic displacement parameters. Except nitrogen N_7_ linked hydrogen atom that was introduced in the structural model through Fourier difference maps analysis, H atoms were finally included in their calculated positions. A final refinement on *F*^2^ with 1645 unique intensities and 86 parameters converged at ω*R*(*F*^2^) = 0.0737 (*R*(*F*) = 0.0282) for 1494 observed reflections with *I* > 2σ(*I*).

**Figure 1**

The molecular structure of the title compound. Displacement ellipsoids are drawn at the 50% probability level.

**Figure 2**

Part of the crystal structure with hydrogen bonds shown as dashed lines.

*Special details*

**Geometry**

All esds (except the esd in the dihedral angle between two l.s. planes) are estimated using the full covariance matrix. The cell esds are taken into account individually in the estimation of esds in distances, angles and torsion angles; correlations between esds in cell parameters are only used when they are defined by crystal symmetry. An approximate (isotropic) treatment of cell esds is used for estimating esds involving l.s. planes.

**Refinement**

Refinement of F^2^ against ALL reflections. The weighted R-factor wR and goodness of fit S are based on F^2^, conventional R-factors R are based on F, with F set to zero for negative F^2^. The threshold expression of F^2^> 2sigma(F^2^) is used only for calculating R-factors(gt) etc. and is not relevant to the choice of reflections for refinement. R-factors based on F^2^ are statistically about twice as large as those based on F, and R- factors based on ALL data will be even larger.
