# Supplementary figures and images for: Crystal structure of 2-(1H-imidazol-4-yl)ethanaminium chloride
Source: Acta Crystallogr E Crystallogr Commun. 2015 Apr 9;71(Pt 5):o301–2. doi: 10.1107/S2056989015006866 (PMC4420062; doi:10.1107/S2056989015006866)

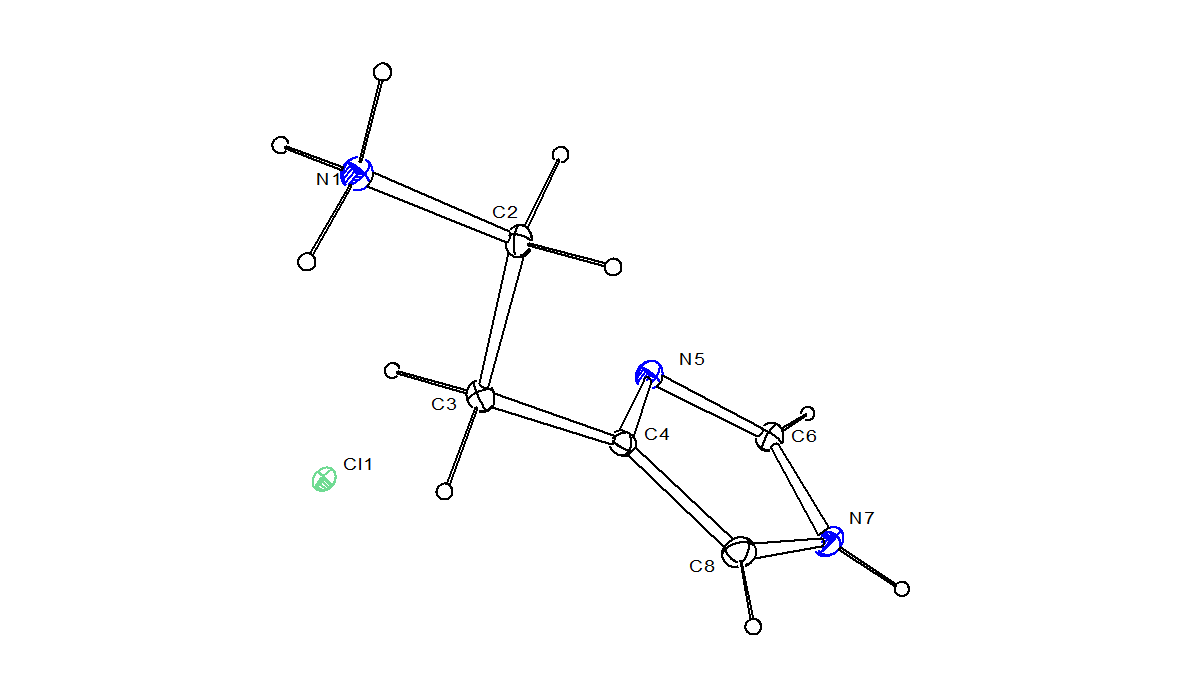

Supplement: Supplementary file 4 [file e-71-0o301-fig1.tif]

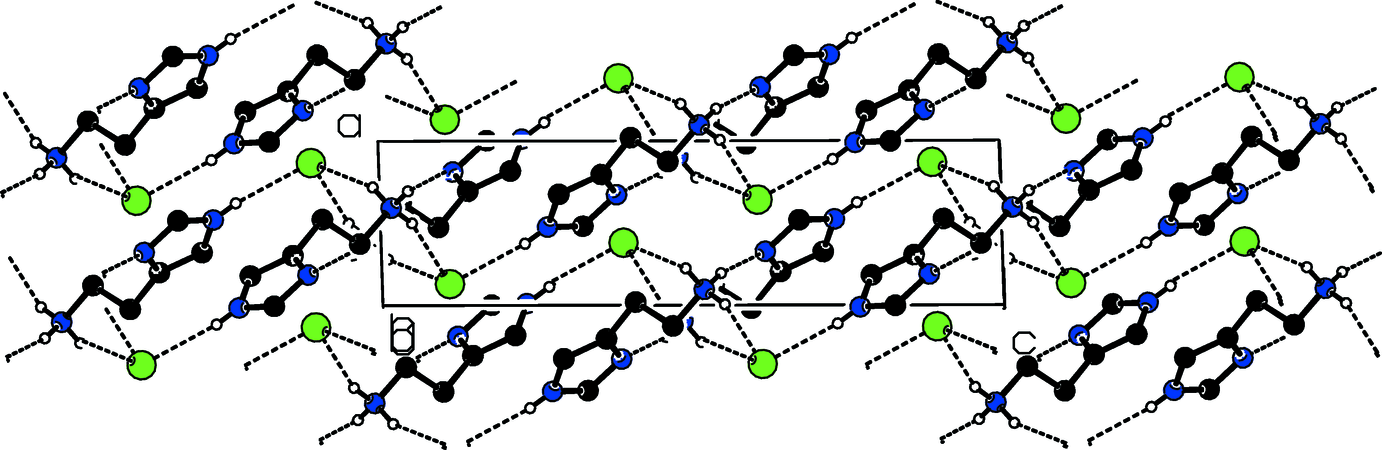

Supplement: Supplementary file 5 [file e-71-0o301-fig2.tif]
